# Supplementary figures and images for: The Dual Role of Oxidants in Male (In)fertility: Every ROSe Has a Thorn
Source: Int J Mol Sci. 2023 Mar 5;24(5):4994. doi: 10.3390/ijms24054994 (PMC10002566; doi:10.3390/ijms24054994)

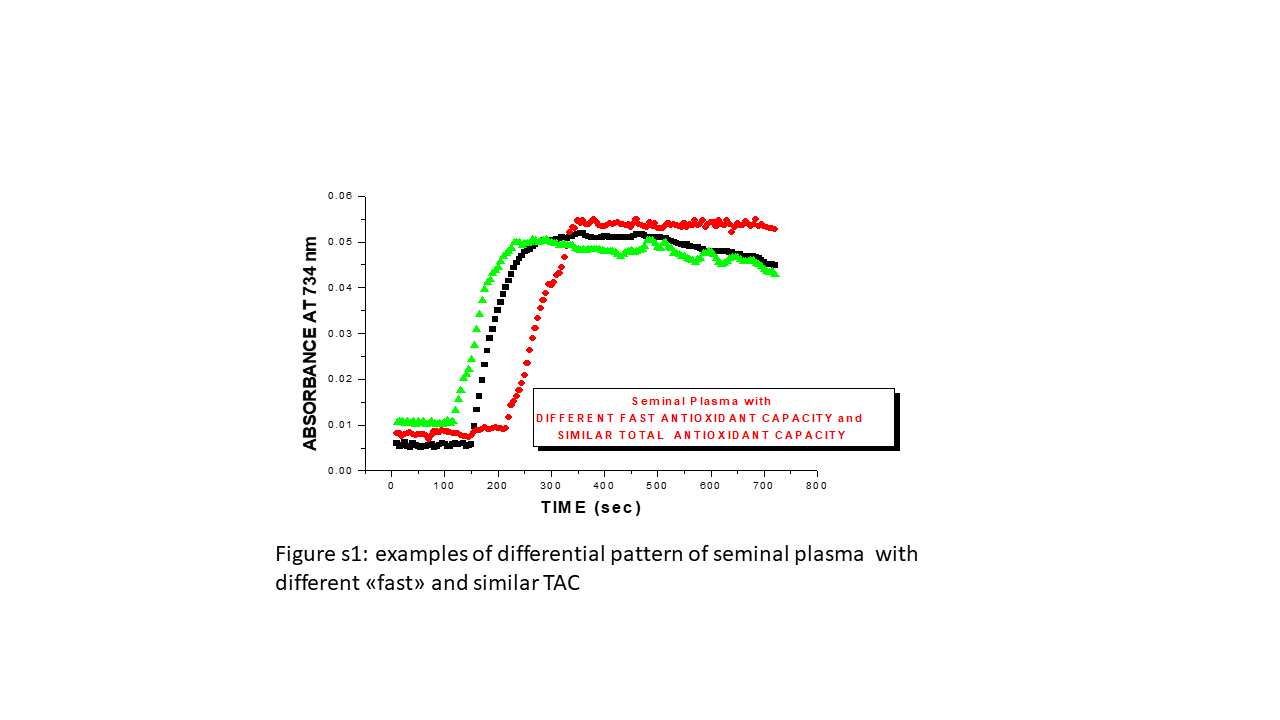

Supplement: Supplementary file 1 [file ijms-24-04994-s001.zip › Diapositiva1.TIF]

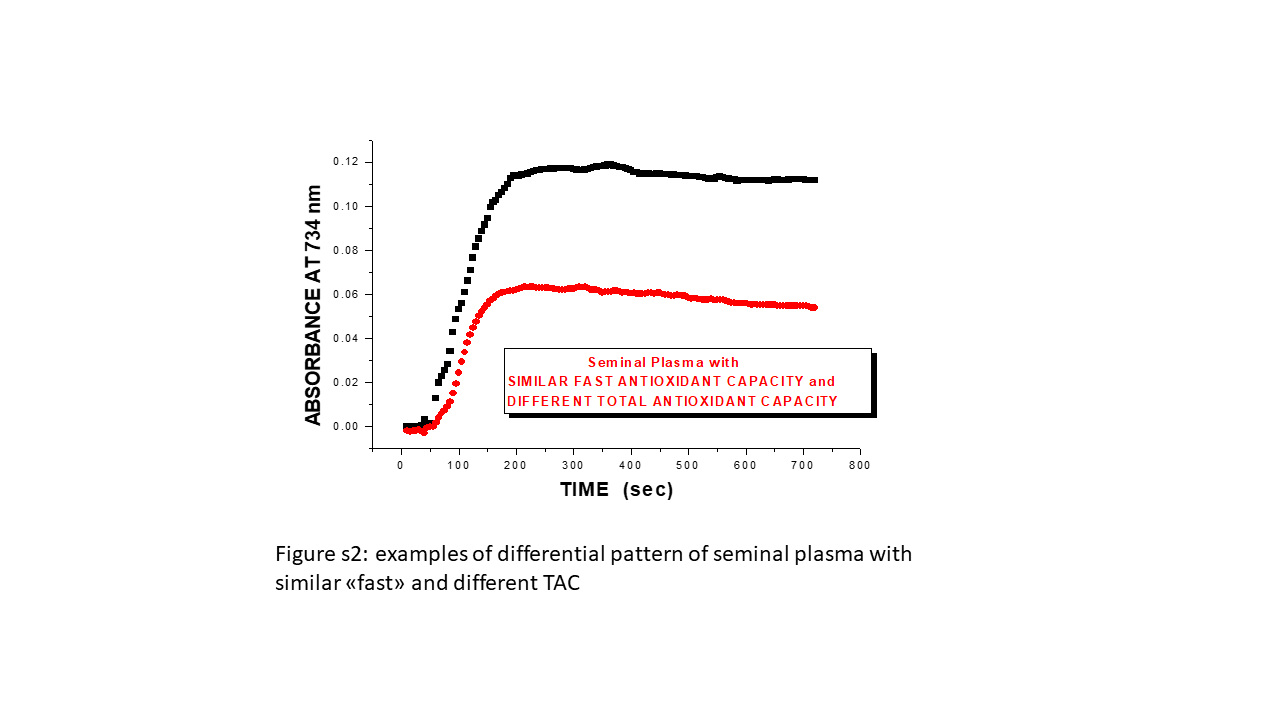

Supplement: Supplementary file 1 [file ijms-24-04994-s001.zip › Diapositiva2.TIF]
